# Around the Hospitals

**Published:** 1894-02-24

**Authors:** 


### Around the Hospitals.

Notice to the Managers of Institutions.—Special space is now reserved for the insertion of notices of hospital meetings and festivals. The Editor requests that all notices may reach The Hospital Office, 428, Strand, at least one week before the date of each meeting.

**The Glasgow Royal Infirmary.**—It is really a most unfortunate circumstance that the inhabitants of Glasgow have not yet seen their way to reducing the large debt of the infirmary beyond a few pounds. This debt now stands at £11,000, a very serious burden for the institution. The question is seriously considered as to reducing the number of beds. This would be a serious pity, for the infirmary is essential to the well-being of the poor of Glasgow and as an educational agent. It shows moreover a determination to be in the front ranks of progress and efficiency. The advantage afforded to female students alone are exceptional. Special days are set apart for them in the dispensary and in the women's wards, besides the usual clinical teaching in the wards. Nursing is now on an excellent footing, and the nurses are comfortably housed in the rearranged home. The convalescent home proves of great benefit, and the gratuitous supply of water to the infirmary, and the maintenance of telephonic communication with the city free of charge by the Water Commissioner and Telephone Company is much appreciated. During the past year 5,197 in-patients and 22,166 out-patients were treated. The total revenue amounted to £34,822 17s., and the total expenditure to £34,346 15s. The cost per occupied bed was £57 16s. 11¼d., the cost per patient £6 0s. 9½d.

**The Essex and Colchester Hospital.**—We regret to learn that the attempt to raise falling annual subscriptions by means of 1,000 circular letters issued on behalf of the hospital has not been successful. The subscriptions show a decrease during the past year, although the church collections and Saturday Fund collections have fallen very little short of previous years. The hospital has, however, been more unfortunate in regard to legacies, none having fallen in. The donations barely reached £100. Under these circumstances it is not surprising that the expenditure exceeds the income, the balance due to the treasurer being £291. The total ordinary expenditure for the year has been £3,439, but we fail to find a statement of the total receipts. The cost per in-patient is given as £4 8s. 10d., the cost per out-patient as 4s. 2½d., drugs and a share of establishment charges being included. This proportion of out-patients is, of course, very large. The in-patients numbered 648, and the out-patients 2,206. The improvements and additions of the year consisted of the painting of two wards and of offices, and the purchase of new sheeting and blankets.

